# Behind Prison Walls: Critical Overview of the Mental Health Trajectories of Children Living With Incarcerated Mothers

**DOI:** 10.7759/cureus.64664

**Published:** 2024-07-16

**Authors:** Priya Prakash, Priyal Khurana, Mayank Gupta, Jayakrishna S Madabushi

**Affiliations:** 1 Psychology, National Institute of Mental Health and Neurosciences, Bengaluru, IND; 2 Psychology, Christ University, Ghaziabad, IND; 3 Psychiatry and Behavioral Sciences, Southwood Psychiatric Hospital, Pittsburgh, USA; 4 Psychiatry, Alabama College of Osteopathic Medicine, Birmingham, USA

**Keywords:** psychological development, prison health, co-living children, incarcerated mothers, child development

## Abstract

Insufficient resources have been identified as a significant factor contributing to delayed development across all domains for children living with their incarcerated mothers. Often lacking extended family support, these children experience environments resembling confinement, devoid of essential cognitive, social, and emotional stimuli crucial for their development. This deprivation can result in substantial educational setbacks and hinder their social integration. This review aims to examine the impact of the prison environment on the development of children residing with their incarcerated mothers. Current research underscores a notable scarcity of comprehensive data on the developmental paths of these children. Some studies suggest that prison nurseries may cultivate positive intergenerational attachments, potentially mitigating the typically low resilience observed in cases of maternal separation. However, while lower-order cognitive functions may not exhibit significant delays, the development of higher-order thinking skills presents more considerable challenges. Addressing the developmental risks faced by children in prison settings is critical, given their heightened vulnerability to systemic neglect. Therefore, prioritizing optimal child development is essential to ensure these children achieve their milestones.

## Introduction and background

René Spitz propounded that babies kept in institutions for care suffer from what he termed as “hospitalism” [1]. He further adopted the term anaclitic depression to explain the phenomenon, which refers to a child’s reaction of grief, anger, and indifference in response to the loss of a loved object (primary caregiver). The recovery is rapid when the loved object is returned within three to five months. However, when prolonged, symptoms of serious deterioration are exhibited, which Spitz referred to as hospitalism. He did studies focusing on infants who were abruptly separated from the primary caregiver when sent to prison. He found that these babies had a developmental decline, lost weight, and withdrew themselves from interacting. He concluded that at least six months of good enough relations between children and mother brings back the equilibrium after the child is reunited [1,2].

There are 97 jurisdictions that allow children to co-reside with their mothers in separate units [3]. The children under the age of six years of age are permitted to reside with their mothers in jail under custody-based settings or if other alternatives are not available. This has led to a surge of incarcerated or in-trial mothers at 1,650 prisoners with 1,867 children [4]. It is critical to underscore that in India particularly, out of the 478,600 prisoners in total, there were 19,913 female prisoners, and in one concentrated state, there were 1,543 incarcerated mothers with 1,779 children [5]. 

The past three decades have observed a drastic surge in overcrowding of prisons. An increase in the total number of prisons exceeding their capacity to hold inmates is seen in prisons in India, Thailand, and Ghana [6]. These alarming trends are comparable to those of other countries, including the United States and many European countries, where the number of inmates exceeds that of other developing worlds. The overall ratio between male and female prisoners globally is 10.9 million to 0.8 million [7]. 

There may not be sufficient cells to house women inmates, and as a result, approximately 90% of the women population reside in general cells [8]. Incarcerated mothers are required to have regular health consultations and counseling services to prepare for themselves and their children. While these needs go unmet, incarcerated mothers indicate a greater risk of mental health issues, with 87.8% reported experiencing significant distress. Prevalent concerns were depression, anxiety, and somatization disorders, with 73.3%, 77.78%, and 82.22%, respectively, ranging between moderate to severe levels [9]. 

Countries in Asia, namely India, Cambodia, and Indonesia, also display an evident rise in the number of females in prisons [10]. The children stay in a separate section of the prison called the prison nursery. The child must be transferred to a surrogate of their choice once they exceed the age limit, or stay under protective custody at the Department of Social Welfare home until they complete their sentence [11]. While the child resides in prison, the state is required to cover all the expenses of food, clothing, and healthcare facilities. 

Despite the standards of normative care outlined by the United Nations, it has been found that these facilities remain fairly limited in the actual scenario [3]. The healthcare facilities provided to the children living in prisons are limited, with no regular check-ups with a pediatrician. The children’s nutritional demands are unmet, as they are served the same meals as the inmates [12]. The literature suggests a wide gap between the proposed policies and facilities and what is delivered inside the prison systems. As these factors contribute to the health conditions of mothers and their children physically and emotionally, understanding these gaps and how they contribute to improving their living conditions is imperative. The current review attempts to analyze the physical, psychological, and social development of children co-living with incarcerated mothers in prisons. An assessment of infrastructural and environmental feasibility is also considered to provide insights and address identified issues.

## Review

Methods

**Figure 1:**
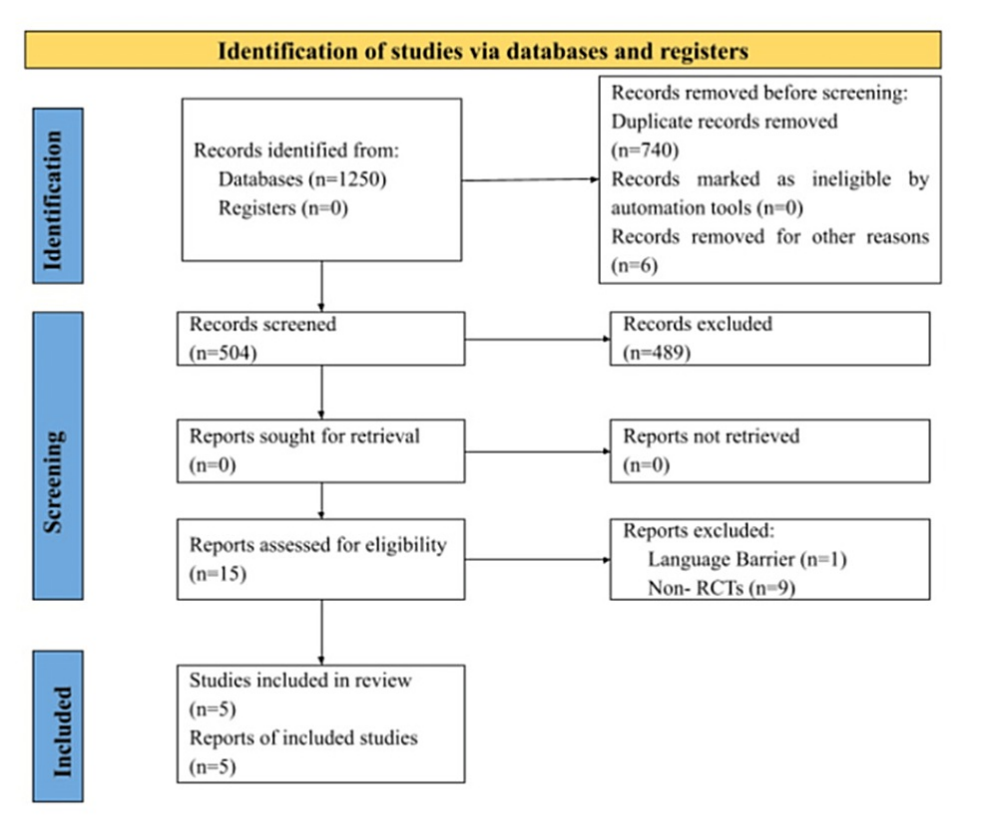
PRISMA 2020 flow diagram for new systematic reviews which included searches of databases and registers only. PRISMA: Preferred Reporting Items for Systematic Reviews and Meta-Analyses, RCT: Randomized control trial.

A literature search was conducted on PubMed, Embase, and Google Scholar, and studies from inception published till February 2024 were included. The search was conducted by authors PP and PK; both reviewers gathered an equal number of studies independently. The key search terms used included “incarcerated mothers,” “prison nurseries,” “incarcerated women,” “mothers in prison,” “co-living children,” “incarcerated mothers,” “children,” “child programs,” and “incarcerated mothers.” A further search included references to all the relevant articles. The inclusion criteria consisted of 1) randomized controlled trials (RCT) in full-text open access and English; 2) studies focusing on issues of incarcerated mothers and their children; and 3) studies emphasizing psychosocial outcomes of children who lived in prison nurseries. The exclusion criteria followed consisted of 1) review articles, meta-analysis, systematic reviews, abstracts, editorials, newspaper articles, conceptual models, and conference abstracts and 2) studies focusing on children of incarcerated fathers. The search strategy provided 504 results that were mutually screened from a vast 1,250 papers, of which five complemented the inclusion criteria and were included in the present review selected by the authors. A summary of the included studies is depicted in Figure 1.

While PK gathered the studies, PP reviewed the study on the basis of the inclusion criteria. Out of 1,250 studies, 740 were eliminated on the basis of duplication, and factors such as language barriers and the lack of full-text access eliminated six studies in the identification stage, leaving 504 studies for the screening process. The papers were further reviewed by MG to further eliminate bias at the identification stage (Figure 1). Papers were reviewed of which 504 studies were narrowed to 15 studies at the final stage of screening with no retrieval. The final 15 papers were further reviewed by PP, PK, and MG to exclude three papers due to language barriers and nine papers as they were not RCTs. The final set of studies was shortlisted into five studies that matched the criterion. To ensure data screening and eliminate further bias, researcher JM conducted an independent supervision of the screening process. Any disagreements pertaining to the inclusion or exclusion of the papers were discussed with respect to their relevance to the sampling criteria.

Results

The key findings of the current review highlight that the mean age of children co-residing with their mothers is 2.8 years and the mean age of mothers is 28 years. The children vary between 0 to 8 years in mothers who raise their children in prisons and up to early adolescence among mothers who avail of prison nursery facilities. A majority (60%) of our sample qualifies from the USA followed by the UK (20%) and Turkey (20%). The average time period of the mother’s incarceration is not mentioned in the above studies. Table 1 provides a brief overview of the countries, sample size, the aim, and the results of each scientific paper. The literature includes studies from the countries: the USA, the UK, and Turkey. The papers under review are between the years 2004 and 2024. Each paper studies the role of prison nurseries in establishing a healthy attachment style between mothers and their children. A majority of the studies focus on interventional models that evaluate the attachment styles of the mother and child which may shape the child’s attachments in subsequent relationships.

**Table 1:** RCTs included in the review highlighting issues faced by incarcerated mothers and their children in prisons and its effects on the child’s development course. BAMBI: Baby and Mother Bonding Initiative; APSQ: Athlete Psychological Strain Questionnaire; RCT: Randomized control trial.

Study	Methodology	Results and implications
Framework: Attachment between mother and child co-residing for at least 1 year; community and maternal risk factors (Goshin, 2014) [13]. Purpose: Compare the patterns of attachment between in-prison residence and brief residence nursery programs.	Design: Interventional design; visitations from Nurse Practitioner (NP) parallel to which children re-entering the community were arranged with caregivers and bi-weekly check-ins to assess the quality of the mother-child relationship and health of both parties. Scales: Strange Situation Procedure; Adult Attachment Interview Sample: 30 females; the number of children, race, and ethnicity unspecified.	1. Both forms of interventions observed an increase in secure attachment between mother and child. 2. Secure maternal attachment styles were lower than the community programs. 3. Despite maternal risk factors, the prison programs displayed an increase in the secure attachment style between the mother and the child. Implications: with an increase in the studies conducted, the focus could shift to reducing the environmental risks and resources to improve the replication and efficacy of the program.
Framework: BAMBI nursing program. Purpose: effectiveness of the out-of-nursery project and the mother-child attachment and competencies of child-rearing among incarcerated mothers (Kwarteng-Amaning et al., 2019) [14].	Design: unconventional prison facilities where incarcerated mothers and children can bond and live together. Exploratory descriptive design Scales: AQSQ questionnaire; Parenting Dimensions Inventory (PDI-S). Sample: 21 children; 41 females (Race: 8 African-American, 12 Hispanic, 19 white, 2 other); Age range: 25Y to 34Y.	1. Mothers reported that they identified their children have insecure attachment styles. 2. Increased care from the family provides a secure attachment in children. 3. Number of children can reduce the amount of nurturing and attachment provided by mothers. 4. Mothers presume that children living in prisons have an insecure attachment style. However, it could be a reflection of the mother’s thoughts and feelings. Implications: Need for improving the quality of parental education. The BAMBI program was reported as effective.
Framework: New beginnings intervention (Byrne et al., 2010) [15].Purpose: to determine the effectiveness of the prison nursery program on the attachment between mothers and their babies.	Design: RCT with 3 phases. Sample: All 7 prisons in UK; 75 dyads; maternal demographics - 68 females; 51 White, 15 Black, 4 Asian and 5 mixed. Infant demographics - 30 males, 45 females. Scales: Parent Development Interview, The Center for Epidemiologic Studies Depression Scale (CES-D), Mother’s Object Relations Scales (MORS).	1. Mothers in the high-risk (clinically significant depressive scores) group displayed a low feeling of warmth when reflecting their bonds with their babies. 2. The behavioral interactions remained stable without significant changes in the intervention group in comparison to the control group. 3. No recorded changes in the reflective functioning and mother-child interaction. 4. Infant’s age and maternal ethnicity played a significant role in maternal object representations. 5. An unprepared separation from their children and a lack of support from the family affects their relationship with their babies. Implications: need for interventions that specifically cater to high-risk dyads; there is a need to observe maternal depression and object relationships longitudinally.
Framework: Prevalence of psychiatric disorders among incarcerated women (Teplin et al., 1996) [16]. Purpose: to evaluate the mental status and development of children co-living with their incarcerated mothers.	Design: Cross-sectional research design. Scales: Prison Experience and Socio-demographic Data Evaluation Form; Beck Depression Inventory (BDI), Beck ANxiety Inventory (BAI), Multidimensional Scale of Perceived Social Support (MDSPS), Childhood Trauma Screening Questionnaire - 53 item version; Denver- II Developmental Screening Test, Kiddie-Schedule for Affective Disorders and Schizophrenia for School Age Children-Present and Lifetime Version- Turkish Version. Sample: 24 mothers and 14 children. No additional demographic details were provided.	1. 1/3rd of the children exhibited developmental delays while the remaining had motor-mental development aligning with their age limit. 2. Children were not receiving treatment for developmental delays. 3. Of the mothers 37.5% had ongoing psychiatric treatment while 70% revealed psychopathology through the measures; 54.2% reported domestic abuse; 45.8% had a history of self-harm and 8.3% reported sexual abuse. 4. Emotional and physical abuse in the mothers.
Framework: Prison nursery interventions and its effects (Nolvi et al, 2023) [17].Purpose: effects of living in prison nurseries between one to 18 months.	Design: Randomized controlled trials Scales: Child development/ behavior problems; Ecological risks Sample:111 children (group 1: 47 preschoolers- co-residing in prisons for 18 months; group 2: 64 preschool children, living in separation); 97 mothers (59 Black, 25 Latina, others- 26); Mean age- 28 in group 1 and 24.8 in group 2	1. Co-residence with mothers in a prison nursery may contribute to resilience against anxious/depressed behavior problems 2. Nursing interventions and regulating environmental risks increased secure attachment in children. 3. Separation affects developmental outcomes. Implications: the caregiving environment, mother’s history of physical and verbal abuse toward the child could be further studied to limit factors affecting development.

Each study has identified the positive effects of developing secure attachments in children despite their mother’s insecure attachment styles. Mothers were screened for mental and physical illness. Both Turkey and the UK have reported maternal depression, anxiety, and substance dependence as risk factors that may impact the child’s development. In Turkey, 1/3rd of the children were reported to have developmental delays and the remaining children reported delays in motor-mental development. Three out of five studies reported familial support outside the prisons played a protective role in mother-child separation. Interventional studies in the USA reported resilience against anxiety/depression in co-residing children with a negative effect on children’s development while they are separated from their mothers. 

Additionally, the literature focuses on the lifestyle, resources, and mental well-being of the children and their incarcerated mothers. 

The reviewed articles have identified that issues persist deeper than the child's attachment to the mother. These issues include administrative discrepancies, lack of awareness, unresolved trauma and mental illness in the mothers, and inadequate social rehabilitation upon release as the primary obstacles to the child's optimal development. Under conditions of adequate healthcare, nutritional meals, infrastructural improvements, and education, it was identified that the child benefits from living with the mother and develops a secure attachment style [13,18]. Administrative inefficiency and the lack of healthcare and childcare facilities are further supplemented by poor policies and interventions in place to reduce recidivism and improve child development.

Discussion 

The present review attempted to analyze the physical, psychological, and social development of children co-living with their incarcerated mothers. Through this review, we identified that there are multiple pitfalls that are not entirely within the control of the mothers but require administrative and environmental support in order to ensure optimal development of their children. It's evident, based on the review of existing scattered literature, that there are many gaps in understanding highly complex issues pertaining to the needs of the infants and children of incarcerated caregivers. The previous literature has identified that there is a strong disparity between the conditions of female and male prisons. Female prisons require hygienic spaces and adequate resources to sustain the health of women; more so in mothers co-living with infants or are pregnant. Due to the rise in female prison population, there is a persistent demand for better facilities and policies. Nonetheless, prison administration displays a vast disparity between the policy and implementation. Prison healthcare, on paper, is expected to provide resources and infrastructure to maintain nutrition, regular screening for mental illness, and routine health checks along with parental education and counseling services. However, prison systems display a harsh reality of outdated policies and inadequate resources. 

The present review aligns with the findings across previous literature. Calls for reforms to move the criminal justice system from retributive to corrective positions should not fail to address potential harms during developmentally critical periods in the mother and child relationship. Poised at the intersection of cutting-edge empirical data on attachment, ethical principles governing the sentencing of pregnant women and mothers of young children, and advocacy for better informed, more just, and more compassionate discretion in the enforcement of expected standards in prisons. The following subsections elaborate on the current review’s findings in alignment with the existing literature. 

Child Development in Prisons and Mental Health Sequelae

The social and cognitive development of the children raised in prisons is not on par with their counterparts who live in mainstream society. While daycare provides an alternative environment for the child for a few hours, it is a temporary fix to a permanent problem. The conducive environment for a child’s social and cognitive development is limited to those few hours a day [19]. Living in prison systems can elicit a spectrum of behavioral and emotional responses, from resilience to anxiety or depression [13]. While mothers feel reassured that their children are present and raised by them in the prisons, they emphasize the lack of conduciveness in the environment, as they display mental and behavioral issues during early childhood [20]. Existing theories such as Spitz's theory were studied in the 1980s by Catan, and reported progressive declining motor and cognitive scores while infants spend their first months in prison nurseries [21]. The changes returned to mean scores within one month of release [13]. The sampled study from Turkey aligns with Catan’s key findings; the mothers have reported that the significant cognitive and motor decline in their children was caused by the lack of timely health screening and nutritional development [22]. However, studies indicate resilience and ability to combat depressive and anxious behavior in children co-living with their mothers. 

State of Prison Nurseries and Childcare Services 

Prison nurseries are understood as the only means to create a mother-child relationship. In a study conducted by Campbell and Carlson in 2012, they focused on the correctional administrator's perspectives of prison nurseries [18]. It was understood that several nurseries lacked the support and facilities the mothers needed to raise their children. Concerning access to healthcare, a study by Paynter et al. in 2022 reported prison systems followed "security over care" systems, the minimum is provided to fulfill the legal demands, and meaningful care is out of reach [23]. Incarcerated mothers have reported that they are provided just as much living space as inmates who live without children or individuals who are not mothers. To add to this constraint is the lack of a separate space for mothers who live with their children in prison nurseries [24]. This difficulty is multiplied if the mother lives with more than one child in prison. The officers believed that creating a space for mothers to raise children would demand money, training, and resources they did not have. Another finding unveiled that officers are unaware of prison nursery systems [18]. While we focus on the nurseries, we can identify that most do not have the resources required for child-rearing. While children grow up with their mothers in prisons, they tend to have a healthy attachment with their mothers through adequate interventions and parental training. However, external pressures can impact the mental health and well-being of the mother, in turn influencing the child [14]. Moreover, in a Turkish study done in 2016, which included 23 children between the age range of 0-66 months, it was observed that children displayed accurate development, and even a higher development than their biological age was exhibited in some cases [22]. This provides a commencement for studying the long-term trajectory of development of these children. 

Emphasis on Maternal Health and Parental Education

Across our concentrated selection of studies, maternal health had substantial importance in the development of the child. Mothers with a high risk of depression and substance usage have a higher chance of neglecting parental responsibilities, feeling overwhelmed by their presumed inadequacy, and reporting that their child’s development is not on par with the expected developmental outcomes [14,25]. The current studies have resulted in positive outcomes through their prison nursery programs and interventions where equal importance was applied to the well-being of the mothers and their children [15]. An improvement in the mothers’ physical and mental well-being had a direct impact on the resilience and emotional regulation of their children [13]. Optimal developmental outcomes of the child cannot be independent of the mother. While the prison environment has consistently been reported as non-conducive to the child’s development, separating the child from the mother has negative consequences across the child’s lifetime; in lieu of maintaining healthy relationships and attachment as well as emotional regulation [16,20]. As for pregnant women in prisons, perinatal care has been identified as important in the physical and cognitive development of the child, and maternal distress at the time of pregnancy has been linked with aberrant brain development, specifically the hippocampal volume and fronto-limbic connectivity. Additionally, it impacts psychopathological interference in later life [17]. 

While mothers have limited power to make decisions within the prison environment, the role of the administration would have a substantial impact on the child’s development. Existing literature substantiates that mothers feel burdened by their inability to make decisions for their children, often caused by restrictions placed by prison correctional officers [26]. The inadequate care and resources provided by the health staff, a lack of timely testing such as ultrasound/sonogram in the name of security, and inability to support breastfeeding, among other discrepancies from the staff, have negative outcomes on the child’s cognitive and social-emotional development [23]. An analysis by National Women’s Law Centers rating these prison systems on prenatal care and family-based treatment alternatives found 21 states receiving D’s and F’s and only Pennsylvania with an A-grade [27]. Across the United States, only 30 states received passing grades. The shortcomings were found in the areas of routine medical check-ups, nutrition, treatment for those with high-risk pregnancies, and HIV screening [28]. 

Administrative Gaps in Upholding Standards 

A recurring theme across papers was the lack of social, governmental, or administrative support that could help integrate mothers and their children into normal lives. Children who are separated from their imprisoned mothers experience multiple issues, including social prejudice, stigma, shame, and the lack of maternal care or support that they deserve [20]. While there is a lack of support for the mothers, they experience a lack of spaces that could address their issues. A study stated that there was difficulty in receiving permission due to the policy and access limitations [13]. In contrast to the prison nursery systems, we can identify that the external environment lacks the conduciveness required to facilitate the child's development. Children residing in prisons are isolated from the realities beyond the prison system [29]. They are separated from their fathers and/or other extended family members. Mothers have identified an impact of the prison environment on the child’s overall development, which they assume might have been caused by the harsh prison conditions [30]. There are constant fights and space-related frustrations among the mothers [19,20]. Only eight prisons across the United States allow incarcerated mothers to keep their infants inside the jails. Often, these infants are handed over to either a relative or a foster home hours after the birth, and it usually takes up to two years for the mother and child to reunite [31].

## Conclusions

The intersection of prison conditions and child development for those co-residing with incarcerated mothers presents complex challenges. Research highlights the adverse effects of separating children from their mothers, including emotional dysregulation and insecure attachment in later years. Despite the benefits of a mother’s presence as the primary caregiver, significant deficiencies remain in the developmental outcomes of children in prison environments. Incarcerated mothers often face health issues such as substance addiction, depression, and anxiety, which impede their parenting abilities. Enhancing mothers' physical and mental health is crucial for active participation in their children's lives, promoting long-term benefits and reducing recidivism. Despite positive responses to prison interventions, inadequate parenting skills and awareness among these mothers negatively impact their children, who depend solely on them. A major issue is the lack of resources and support within prisons. Mothers frequently report insufficient facilities for raising their children, and government policies rarely address their specific needs. Additionally, children often lack connection to the outside world due to limited support from extended families and poor community integration. There is a significant gap in scientific research on the developmental outcomes of children co-residing with incarcerated mothers, especially in the Southeast Asian countries. Addressing these gaps and improving prison environments are crucial for enhancing these children's quality of life and long-term development.
